# Electromagnetic-Wave Absorption Properties of 3D-Printed Thermoplastic Polyurethane/Carbonyl Iron Powder Composites

**DOI:** 10.3390/polym14224960

**Published:** 2022-11-16

**Authors:** Yinsong Zheng, Yan Wang

**Affiliations:** School of Materials Science and Engineering, Wuhan Institute of Technology, Wuhan 430074, China

**Keywords:** TPU, CIP, fused deposition modeling, 3D printing, absorbing structure

## Abstract

To develop a composite filament with an electromagnetic-wave-absorbing function suitable for 3D printing, we combined thermoplastic polyurethane (TPU) as the matrix material and carbonyl iron powder (CIP) as the absorbing agent to prepare TPU/CIP composites by melt blending. The composites passed through a single-screw extruder to obtain a filament with 2.85 mm in diameter. Different absorber structures were printed using fused deposition modeling, and their absorption properties were tested using the bow method. The results showed that by increasing CIP content, the electromagnetic-wave absorption performance gradually improved, while the mechanical properties substantially decreased. When the mass fraction of the CIP was 60%, the TPU/CIP composite showed good absorption properties and could be prepared into a filament that met the requirements for fused deposition modeling. Simulation results of plate-wave-absorption performance showed that, when the plate thickness was 3 mm, the minimum reflection loss was −21.98 dB, and the effective absorption bandwidth (for reflection loss below −10 dB) was 3.1 GHz (4.55–7.65 GHz). After the TPU/CIP composite was printed into honeycomb, pyramid, and other absorber structures, the absorption performance was further improved. For a structure printed with a gradient-wall honeycomb structure at 3 mm thickness, the effective absorption bandwidth was 4.64 GHz (8.48–13.12 GHz), and the minimum reflection loss was −36.69 dB. The effective absorption bandwidth of the pyramid structure reached 15.88 GHz (2.12–18 GHz), and the minimum reflection loss was −49.75 dB.

## 1. Introduction

The widespread application of electronic devices has brought great convenience to society, but it also causes electromagnetic radiation pollution. This type of pollution has become the fourth largest source of pollution after water, air, and noise pollution [1,2], and it can be mitigated through the use of absorbing materials. Radar stealth aircraft and military facilities also use such absorbing materials widely. Therefore, extensive research is being conducted on functional materials with excellent electromagnetic-wave absorption properties.

Electromagnetic-wave-absorbing functional materials are typically composed of a polymer matrix and electromagnetic-wave absorber. Common absorbing materials are divided into electrical-loss-absorbing agents (e.g., graphene, conductive carbon black, carbon nanotubes) and magnetic-loss-absorbing agents (e.g., ferrite, metal powder) [3,4,5,6,7]. In particular, carbonyl iron powder (CIP) is widely used in composites that absorb magnetic losses, owing to its excellent magnetization effect and good interfacial interaction with polymers [8]. Bahri-Laleh et al. [9] prepared various CIP/polyaniline composites for electromagnetic-wave absorption with different mass ratios by in situ polymerization of polyaniline and CIP in aqueous solution. Their results showed that when the CIP/polyaniline mass ratio increased from 3:1 to 6:1, the minimum reflection loss (RL) improved from −14.3 to −21.0 dB; however, when the CIP mass ratio increased to 10:1, the minimum RL was degraded to −15.5 dB. Therefore, excessive CIP content may attenuate the absorption performance. Gao et al. [10] prepared CIP/polyvinyl chloride (PVC) composites by melt blending to produce CIP/PVC monolayer and PVC–CIP/PVC multilayer functional materials for electromagnetic-wave absorption by hot pressing, and studied their absorption properties. The effective absorption bandwidth (EAB) of the PVC–CIP/PVC alternating multilayer reached 4.7 GHz, and the minimum RL reached −29.8 dB for two alternating layers and a sample thickness of 2 mm. Peng et al. [11] fabricated composites of ferrite with thermoplastic polyurethane (TPU) and found that when the mass fraction of ferrite reached 88.9%, the composite material reached an RL of −20 dB in 1–2 GHz. In general, high CIP content is necessary for composites with excellent electromagnetic-wave absorption performance, but this degrades the mechanical properties and processability. Instead of increasing the content of the absorbing agent to increase the absorbing performance, the structure and impedance matching of the absorbers can be enhanced.

Conventional manufacturing methods are unsuitable for preparing absorbers with complex structures and mechanical bearing functions. Alternatively, 3D printing technology provides outstanding advantages for the rapid manufacturing of complex 3D absorbers. In addition, it allows for the modification of the design at any time according to the application requirements. Given the layered manufacturing, a gradient change in the material impedance of each layer can be obtained to improve impedance matching and perform integrated forming and manufacturing of a broadband absorber [12]. Moreover, a 3D-printed absorbing structure can be used to verify the simulation results of absorbing performance and for the optimal design and manufacture of absorbing structures. Similarly, fused deposition modeling (FDM) is widely used because of its safety, environmental friendliness, wide availability of materials, high integration, unrestricted product shape, and low cost [13]. In a previous study using conductive acrylonitrile butadiene styrene as an absorbing material, we printed samples with various microstructures via FDM. The EAB of a printed wood-pile structure with a thickness of 3.5 mm reached 5.43 GHz [14].

TPU has high strength, good flexibility, corrosion resistance, aging resistance, wear resistance, ease of processing, and high filling capacity. Thus, we selected TPU in this study as the matrix material, and CIP as the absorbing agent, to prepare composites with an electromagnetic-wave absorption function. The composites were assembled using FDM to obtain structures that provide excellent absorbing performance. Hence, we introduce an effective method for optimizing the design and manufacturing of electromagnetic-wave-absorbing structures with broad application prospects.

## 2. Materials and Methods

### 2.1. Materials

TPU (TPU1195A; density, 1.14 g/cm^3^; elastic modulus, 52 MPa; elongation at break, 430%; melt flow index, 65 g/10 min) was purchased from BASF (Ludwigshafen, Germany). CIP (BD-MZ-II, Table 1) was obtained from Wuhan Magnetoelectric (Wuhan, China), and hyperbranched resin (C100) was supplied by Wuhan Hyperbranched Resin Technology (Wuhan, China).

### 2.2. Preparation of TPU/CIP Composites

TPU was dried in an oven at 80 °C for 4 h. Then it was melt-blended with CIP and hyperbranched resin C100 (used as flow modifier) using a mini-mixer (QE-70A; Wuhan Qien Technology Development, Wuhan, China). Blending was performed for 7 min at 190 °C and a rotor speed of 30 rpm. The obtained composites were labeled as P-30, P-40, P-50, P-60, P-70, and P-80 according to the specifications listed in Table 2.

### 2.3. Compression Molding of TPU/CIP Composites

Samples for measuring electromagnetic properties were molded using a hot press (R-3221; Wuhan Qien Technology Development, Wuhan, China). CIP/TPU composite pellets were placed in the mold and pressed for 15 min at 205 °C under 10 MPa. The mold was then cooled, and a concentric annular-shaped sample of 3 × 7 × 2 mm (inner diameter × outer diameter × thickness) was obtained.

### 2.4. Injection Molding of TPU/CIP Composites

Samples for measuring mechanical properties were prepared using a microinjection molding machine (M-1200; Wuhan Qien Technology Development, Wuhan, China). The injection molding conditions were temperature of 215 °C, clamping time of 8 s, injection time of 12 s, and cooling time of 5 s. The dumbbell-shaped samples had dimensions of 75 × 10 × 2 mm (gauge length of 25 mm).

### 2.5. Filaments of TPU/CIP Composites 

The 3D printing filament was prepared in a single-screw extruder (TP-07; Dongguan Songhu Plastic Machinery, Dongguan, China). The first, second, and third temperature zones of the extruder were set to 185, 210, and 205 °C, respectively. The rotation speeds of the screw and tractor were set to 900 and 350 rpm, respectively. The filament diameter was controlled within 2.75–2.95 mm.

### 2.6. FDM Printing

**Figure 1:**
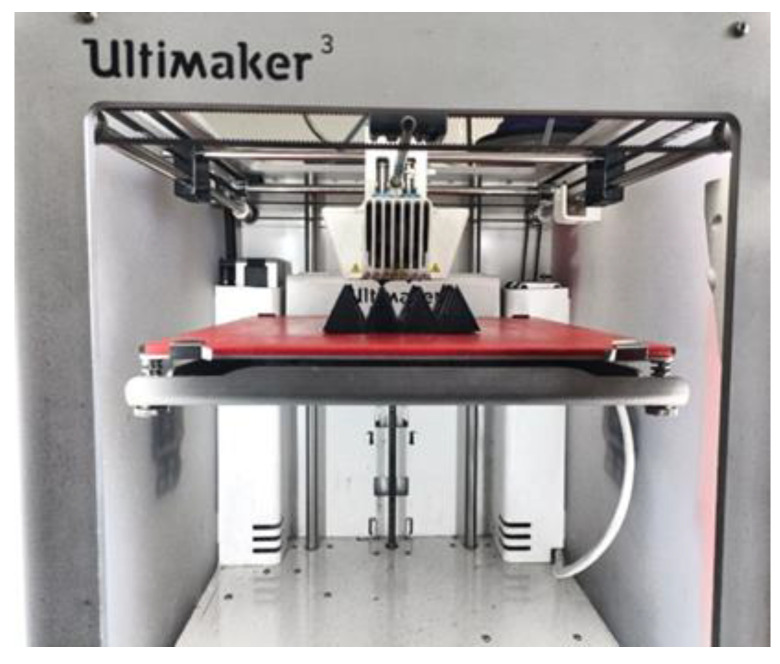
Photograph of 3D printer.

FDM was performed using an Ultimaker 3 printer (Ultimaker, Utrecht, The Netherlands). For printing, we set an initial printing speed of 30 mm/s, nozzle temperature of 205 °C, and build plate temperature of 90 °C. A photograph of the 3D printer is shown in Figure 1.

### 2.7. Testing and Characterization

The tensile properties of the samples were tested using a universal testing machine (5960; INSTRON, Boston, MA, USA) at a strain rate of 50 mm/min according to the GBT1040.1-2018 standard. The dielectric and magnetic permeability parameters of the TPU/CIP composites at 0.3–18 GHz were tested using a vector network analyzer (N5224A; Keysight Technologies, Santa Rosa, CA, USA) by the coaxial method. The electromagnetic-wave RL performance of the printed samples was evaluated using the bow method, with the vector network analyzer in 2–18 GHz. The morphology of the CIP/TPU composite samples was observed through scanning electron microscopy (SEM) (JSM-5510 LV; JEOL, Tokyo, Japan) at an accelerating voltage of 20 kV. The samples were cryofractured in liquid nitrogen, and the fractured surfaces were coated with a layer of gold in a vacuum chamber before SEM visualization.

## 3. Results and Discussion

### 3.1. Effect of CIP Content on Mechanical Properties of TPU/CIP Composites

**Figure 2:**
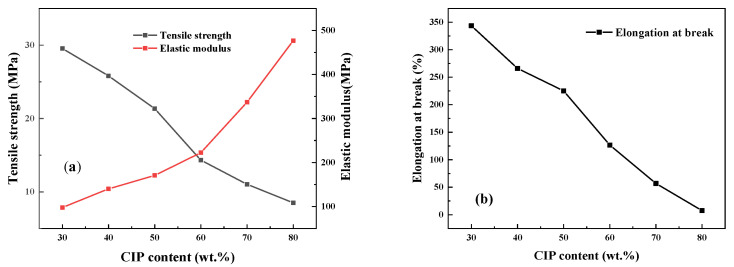
Effect of CIP content on elastic modulus and tensile strength (**a**), and elongation at break (**b**), of TPU/CIP composites.

As shown in Figure 2, by increasing CIP content, the elastic modulus of the TPU/CIP composites gradually increased, but the tensile strength and elongation at break decreased substantially. Being a rigid particle, the modulus of CIP is much larger than that of TPU. Thus, the elastic modulus of the TPU/CIP composites increased with increasing CIP content. Meanwhile, stress concentration occurred owing to the CIP particles, which reduced the tensile strength and toughness of the composites. 

**Figure 3:**
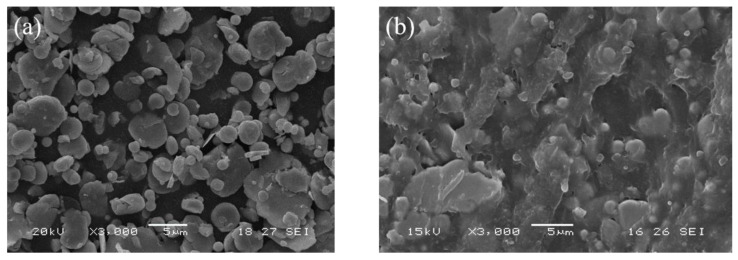
SEM image of CIP (**a**) and sample P-60 (**b**).

Figure 3a,b show SEM images of CIP and sample P-60, respectively. The CIP particles are spherical or flaky, as shown in Figure 3a. Several CIP particles are observed in Figure 3b, and almost no CIP agglomerations are observed, indicating that the composite blend is uniform under the strong shear stress exerted by the mixer rotors.

### 3.2. Effect of CIP Content on Electromagnetic Properties of TPU/CIP Composites

**Figure 4:**
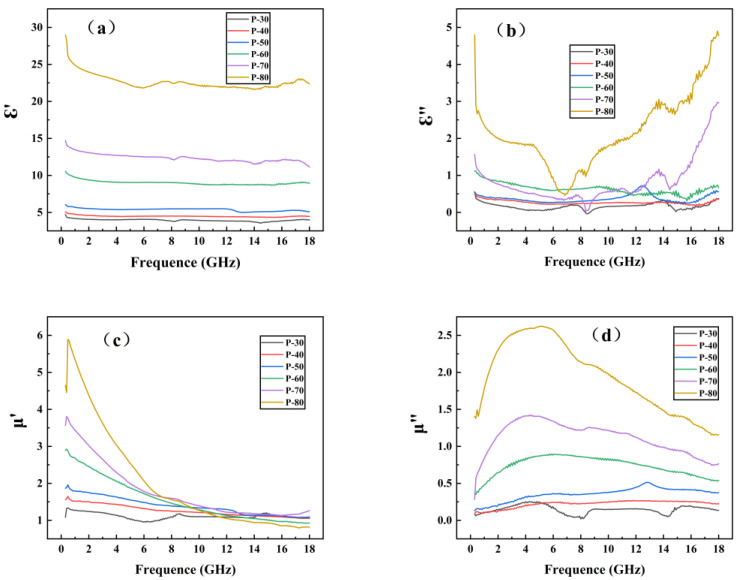
Real (**a**) and imaginary (**b**) parts of dielectric constant and real (**c**) and imaginary (**d**) parts of magnetic permeability obtained from TPU/CIP composites.

Figure 4 shows the dielectric constant and permeability of TPU/CIP composites with different CIP contents, measured using the coaxial method in 0.3–18 GHz.

As shown in Figure 4a,b, from P-30 to P-80, the real ($\varepsilon^{\prime }$) and imaginary ($\varepsilon^{\prime \prime }$) parts of the dielectric constant of the composite increased with increasing CIP content at each frequency, indicating that CIP had a certain dielectric loss capability [15,16]. With increasing CIP content, better CIP dispersion was achieved, thereby improving the dielectric loss performance.

The real part ($\mu^{\prime }$) of permeability reflects the electromagnetic energy storage capacity, and its imaginary part ($\mu^{\prime \prime }$) reflects the electromagnetic energy loss and dissipation capacity, which increased with increasing CIP content. As the frequency increased, $\mu^{\prime }$ gradually decreased, whereas $\mu^{\prime \prime }$ first increased and then decreased. Generally, magnetic losses are caused by exchange resonance, natural resonance, and eddy-current effects. The magnetic loss caused by the eddy-current effect varies with frequency, and the magnetic permeability of the material fluctuates within a certain constant interval. Therefore, the eddy-current loss is the major contributor to magnetic loss [17].

**Figure 5:**
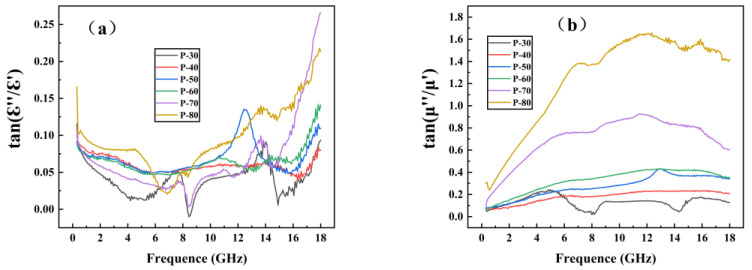
Dielectric (**a**) and permeability (**b**) loss tangents of composite samples.

Figure 5 shows the dielectric loss tangent ($\tan\;\varepsilon_{r}=\varepsilon^{\prime \prime }/\varepsilon^{\prime }$) and magnetic loss tangent ($\tan\;\mu_{r}=\mu^{\prime \prime }/\mu^{\prime }$) of the composite samples. The value of $\tan\;\mu_{r}$ exceeded that of $\tan\;\varepsilon_{r}$, indicating that the magnetic loss provided by CIP played a dominant role in electromagnetic-wave absorption. 

### 3.3. Simulation of Absorption Properties of TPU/CIP Composites

According to the transmission-line theory [18], the reflectivity of a material can be calculated as

(1) $$Z_{r}=\eta_{0}\sqrt{\frac{\mu_{r}}{\varepsilon_{r}}}\tanh\left(\frac{j2\pi fd}{c}\sqrt{\mu_{r}\varepsilon_{r}}\right)$$

(2) $$\varepsilon_{r}=\varepsilon^{\prime }-j\varepsilon^{\prime \prime }\;$$

(3) $$\mu_{r}=\mu^{\prime }-j\mu^{\prime \prime }$$

(4) $$RL=20lg\left|\frac{Z_{r}-\eta_{0}}{Z_{r}+\eta_{0}}\right|$$

where $\eta_{0}$ is the characteristic impedance of free space ($\eta_{0}=\sqrt{\mu_{0}/\varepsilon_{0}}=1$), $Z_{r}$ is the input impedance of the material, f is the frequency of the electromagnetic wave, c is the speed of light, d is the thickness of the material, and $\varepsilon_{r}$ and $\mu_{r}$ are the permittivity and permeability of the material, respectively, which are calculated from the real and imaginary parts of the permittivity and permeability by using Equations (2) and (3).

The RL of the material can be simulated using Equation (4) as the ratio of reflected to incident wave power. A lower RL indicates stronger electromagnetic-wave absorption by the material. The absorption properties of materials are mainly determined under two basic conditions. First, the incident electromagnetic wave can enter the material instead of reflecting back, which requires the material and air to satisfy impedance matching conditions. Second, the electromagnetic waves inside the material can quickly and completely achieve the attenuation loss, which requires the material to have highly efficient attenuation characteristics for electromagnetic waves.

**Figure 6:**
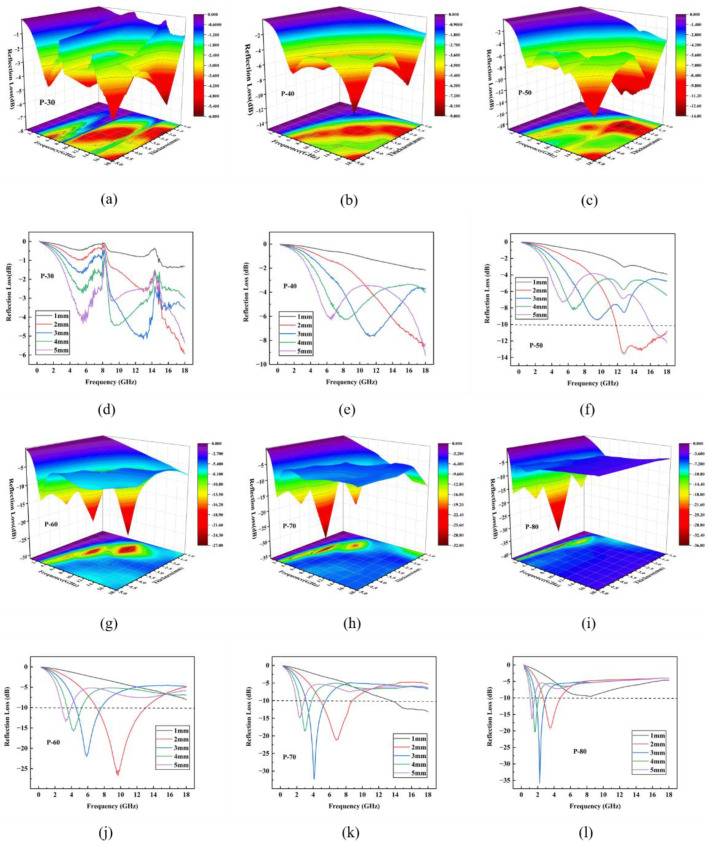
Calculated RL spectra in three (**a**–**c**,**g**–**i**) and two (**d**–**f**,**j**–**l**) dimensions indicating simulated electromagnetic-wave absorption performance of plate samples P-30 (**a**,**d**), P-40 (**b**,**e**), P-50 (**c**,**f**), P-60 (**g**,**j**), P-70 (**h**,**k**), and P-80 (**i**,**l**) for plate thicknesses of 1–5 mm.

Figure 6 shows the simulated values of the sample thicknesses of composite plates with different CIP mass fractions. In 0.3–18 GHz, an increasing CIP mass fraction gradually deepened the RL peak of the TPU/CIP composite, indicating that the RL gradually decreased with more CIP content within a certain range. As the thickness of the composite plate increased, the RL peak gradually moved towards the low-frequency band. This phenomenon may be explained by the quarter-wavelength cancellation model [19]:

(5) $$d_{m}=\frac{nc}{4f_{m}\sqrt{\left|\mu_{r}\varepsilon_{r}\right|}}\;(n=1,\;3,\;5,\;\ldots )$$

where *d_m_* is the thickness of material and *f_m_* is the frequency of the electromagnetic wave. For thickness *d_m_* of the material, a RL peak is obtained at *f_m_*.

Table 3 shows that the minimum RL for samples P-30 and P-40 was −5.93 and −9.26 dB, respectively. The EAB for all the simulated thicknesses of these samples was zero, indicating poor absorption properties. With increasing CIP content, sample P-50 showed a wider EAB of 6.19 GHz, but the approximate RL was only −13 dB. The RL of sample P-60 increased to –26.75 dB while retaining the EAB. Despite the increased RL of samples P-70 and P-80, their EAB was substantially reduced.

Overall, when the CIP content reached a certain value, further increasing the CIP content did not notably improve the absorption performance of the TPU/CIP composite. Considering electromagnetic-wave absorption, mechanical and processing properties of the TPU/CIP composites, P-60 was selected for FDM to print absorbers with different structures for evaluation.

### 3.4. Electromagnetic-Wave Absorption Properties of 3D Printed TPU/CIP Structures

**Figure 7:**
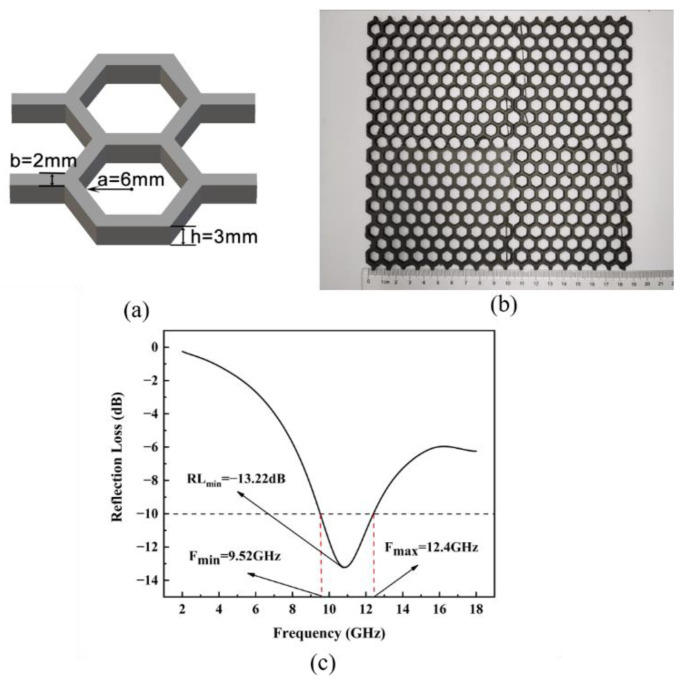
Diagram of straight-wall honeycomb structure (**a**). Printed structure (**b**), and its RL spectrum (**c**).

**Figure 8:**
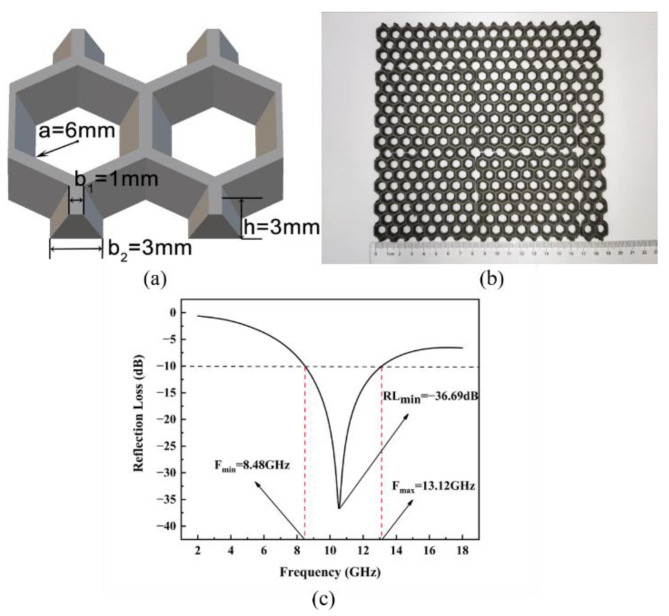
Diagram of gradient−wall honeycomb structure (**a**). Printed structure (**b**), and its RL spectrum (**c**).

**Figure 9:**
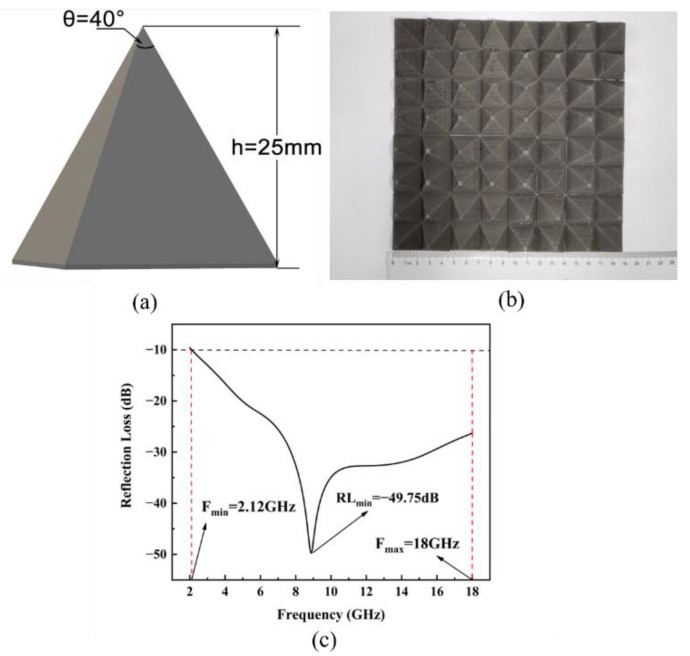
Diagram of pyramid structure (**a**). Printed structure (**b**), and its RL spectrum (**c**).

The P-60 composite was assembled into filaments 2.85 mm in diameter through a single-screw extruder, and printed by FDM into the three kinds of electromagnetic-wave-absorbing structures: straight-wall honeycomb, gradient-wall honeycomb, and pyramid. The absorption properties of each structure were tested using the bow method, as shown in Figure 7, Figure 8 and Figure 9.

#### 3.4.1. Straight-Wall Honeycomb Structure

Honeycomb structures are widely used in electromagnetic-wave absorbers and resemble the structures in nature. When electromagnetic waves enter the hexagonal honeycomb hole, the incident wave is reflected and absorbed repeatedly by the inner walls, thus consuming the wave energy for absorption. The conventional honeycomb absorber is fabricated by impregnating a honeycomb core wall of aramid paper with an absorbing slurry or by filling the honeycomb core hole with absorbing foam [14]. However, this fabrication method is complex, hinders product quality control and results in poor mechanical properties. On the other hand, honeycomb structures with precise dimensions and excellent mechanical properties can be conveniently manufactured using 3D printing. Figure 7 shows a 3D-printed honeycomb structure with an inner diameter of 6 mm, wall thickness of 2 mm, and height of 3 mm. The minimum RL of the honeycomb structure was −13.22 dB, and the EAB was 2.88 GHz. Its absorption performance was worse than that of the simulated 3 mm plate, but a lightweight structure was obtained. Compared with the plate sample of the same thickness, the weight of the honeycomb sample decreased by approximately 70%.

#### 3.4.2. Gradient-Wall Honeycomb Structure

To further improve the electromagnetic-wave absorption performance of the straight-wall honeycomb structure, we designed gradient walls for printing, as shown in Figure 8. The gradient-wall honeycomb structure was derived from a straight-wall honeycomb, and the inner wall was set as the gradient slope. We set the inner diameter of the honeycomb to *a* = 6 mm, the upper inner wall to *b*_1_ = 1 mm, the lower inner wall to *b*_2_ = 3 mm, and the honeycomb height to *h* = 3 mm. The reflectivity measurement showed that the minimum RL reached −36.69 dB, and the EAB reached 4.64 GHz. Compared with the straight-wall honeycomb with the same inner diameter and thickness, the absorption performance of the gradient-wall honeycomb structure was greatly improved and even surpassed the performance of the simulated plate with the same thickness.

#### 3.4.3. Pyramid Structure

As shown in Figure 9, the height of the pyramid structure was 25 mm, and the vertex angle was 40°. The corresponding minimum RL was −49.75 dB, and the EAB was 15.88 GHz, covering the entire range of radar S, C, X, and Ku bands. Among the evaluated absorbers, the pyramid structure showed the best impedance matching because the top of the structure had an impedance close to that of air, thus promoting the absorption of electromagnetic waves by the structure. In addition, the stable interface formed by the magnetic material enhanced absorption. These two factors greatly enhanced electromagnetic-wave attenuation.

Table 4 lists the electromagnetic-wave absorption properties of composites based on CIP as the absorbing agent reported in recent studies, and the properties determined in our study.

## 4. Conclusions

The electromagnetic-wave absorption properties of the TPU/CIP composites gradually improve with increasing CIP content up to a certain concentration. Meanwhile, the tensile strength and elongation at the break of the TPU/CIP composite decreases. The composite with 60 wt.% CIP achieves the best overall properties, being suitable for FDM. SEM images show that the CIP particles are uniformly dispersed in the TPU matrix. The absorption performance of a printed gradient-wall honeycomb structure is better than that of a straight-wall honeycomb structure, reaching a minimum RL of −36.69 dB at a thickness of 3 mm and EAB of 4.64 GHz. The EAB of the printed pyramid structure covered the band of 2.12–18 GHz, and its minimum RL reached −49.75 dB, showing the best applicability as an FDM-printed electromagnetic-wave absorber among the evaluated structures.

## Figures and Tables

**Table 1:** Basic properties and electromagnetic parameters of BD-MZ-II CIP.

Particle Size (µm)	Tap Density (g/cm^3^)	Electromagnetic Parameters (Part of Frequencies)				
		Frequency (GHz)	ℇ′	ℇ″	*μ*′	*μ*″
2–10	2.4–3.3	2	25.21	0.71	4.91	2.46
		8	24.32	0.31	1.77	2.49
		18	26.41	0.26	0.51	1.21

**Table 2:** Specifications of TPU/CIP composites.

**Sample**	**TPU (wt.%)**	**CIP (wt.%)**	**C100 (wt.%)**
P-30	69	30	1
P-40	59	40	1
P-50	49	50	1
P-60	39	60	1
P-70	29	70	1
P-80	19	80	1

**Table 3:** Electromagnetic-wave absorption properties of samples P-30–P-80.

Sample	Minimum RL (dB)	Maximum EAB (GHz) (RL < −10 dB)
P-30	−5.93	0
P-40	−9.26	0
P-50	−13.59	6.19
P-60	−26.75	5.93
P-70	−32.34	2.31
P-80	−35.84	1.15

**Table 4:** Performance comparison of the electromagnetic-wave-absorbing composites and structures.

Materials	CIP Content	RL Test	RL_min_ (dB)	EAB (GHz)	Study
CIP/polyurethane	80 wt.%	Experiment	−11.6	2.5	[20]
CIP/methacrylic	70 wt.%	Simulation	−22	6.0	[21]
CIP/polyimide	60 wt.%	Experiment	−33	2.5	[22]
Flakey CIP/methyl vinyl silicone rubber	50 vol.%	Simulation	−26.1	3.0	[23]
CIP/epoxy resin	40 wt.%	Experiment	−20.8	2.6	[24]
CIP/melt adhesive	30 vol.%	Experiment	−12	7.2	[25]
CIP/TPU	60 wt.%	Experimental using gradient-wall honeycomb structure	−36.7	4.6	This study
CIP/TPU	60 wt.%	Experimental using pyramid structure	−49.7	15.8	This study
